# Protein and fat intake impact on growth of primary school girls in Kerman, Iran

**DOI:** 10.1038/s41598-024-66001-4

**Published:** 2024-07-03

**Authors:** Nooshin Jannati, Mohammad Reza Mahmoodi, Leila Azadbakht

**Affiliations:** 1 Department of Community Nutrition, School of Nutritional Sciences and Dietetics, Tehran University of Medical Sciences (TUMS), Tehran, Iran; 2 Physiology Research Center, Institute of Neuropharmacology and Department of Nutrition, Faculty of Public Health, Kerman University of Medical Sciences, Kerman, Iran

**Keywords:** Quality, Quantity, Protein, Fat, Anthropometric indices, Student, Kerman, Paediatric research, Nutrition

## Abstract

The school age period is characterized by significant physical and intellectual growth, necessitating the monitoring of macronutrient intake and its impact on weight and height. The objective of this study is to investigate the association between the quality and quantity of protein and fat consumption with anthropometric indices in primary school girls in Kerman. This cross-sectional analysis was conducted on primary school girls aged 6–12 (n 330) from ten schools in Kerman, Iran. A validated and reliable dish-based 185-item food frequency questionnaire was used. We calculated the amount of proteins and fats as the percentage of daily calories and grams per day. Plant-based and animal-based proteins were used to assess the protein quality. To assess the fat quality, we considered trans fatty acids (TFA), cholesterol, vegetable oils, solid vegetable oils, animal oils, omega-6 Polyunsaturated fatty acids (PUFAs), omega-3 PUFA, and (PUFAs + Monounsaturated fatty acids (MUFAs))/Saturated fatty acids (SFAs), PUFAs/SFA, and (MUFA + PUFA)/(SFA + TFA). There was a significant positive association between height-for-age z-score (HAZ) and plant protein (p < 0.001) and vegetable oils (p = 0.038) after adjustment. In higher tertiles of animal protein, weight-for-age z-score (WAZ) (p = 0.024) were significantly higher. A significant positive association was observed between omega-3 PUFA and mid-upper arm circumference (MUAC) (p = 0.039) and BMI-for-age z-score (BAZ) (p = 0.016). Our study emphasizes the importance of monitoring protein and fat intake in primary school girls for optimal growth. Positive associations were found between plant-based protein, vegetable oils and HAZ, as well as animal protein and WAZ, highlighting the impact of protein quality on growth indicators.

## Introduction

Child growth is globally recognized as a key indicator of physical well-being in children^1^. Anthropometric indices such as weight, height, body mass index (BMI), and mid-upper arm circumference (MUAC) used to assess growth patterns and body composition in children and adolescents^2^. Overweight, obesity, thinness, and stunting are the most common nutritional and developmental disorders in children and adolescents with significant implications for their health and well-being^3–6^. According to weight-for-age, height-for-age, and weight-for-height, Kerman has malnutrition rates of 6.06, 5.58, and 75.9%, respectively, with malnutrition being more prevalent in girls than in boys^7^. Therefore, early identification and treatment of childhood developmental abnormalities should be considered.

Diet plays an important role in every child’s overall health and influences their development during chronic illnesses^8^. Research on elementary and high school students has shown that obesity and less-than-optimal child development outcomes are risk factors for chronic illnesses that are inversely correlated with low nutrition quality^9,10^. Hence, it is crucial to assess the quality of children's diets. Among macronutrients, proteins and fats are essential for energy and the expected growth process^11^. Studies have revealed that the type and quality of foods have a variety of impacts on body weight in addition to the quantity of each macronutrient, such as carbohydrates, proteins, and fats^12^.

In a previous study on Iranian children, lower protein and fat intake were associated with a higher risk of being underweight, while higher protein intake was associated with a higher risk of being overweight^13^. However, Pourhashemi et al. found no significant association between macronutrient intakes and anthropometric indices in primary school children^14^.

It is essential to monitor both the quantity and quality of macronutrients consumed by children to evaluate the quality of their diet and understand how it affects their weight and height status. The diet and health of girls are particularly important as they will become mothers and have a significant influence on their children's well-being. To the best our knowledge, this is the first study to assess the association between the quality and quantity of protein and fat intake with anthropometric indices among primary school girls in Kerman, Iran.

## Method

### Study design and subjects

Cross-sectional data were collected from 330 primary school girls in Kerman, Iran. Kerman is located in the southeast of Iran, with the administrative center in the city of Kerman. It is Iran's largest province and covers nearly 11 percent of the country's territory. The population of the region is about 3 million people (ranking 9th in the country). To calculate the required sample size for this study, the mean and standard deviation of BMI from a cross-sectional study conducted on 7–11-year-old Iranian children were used (Mean ± SD = 16.0 ± 2.9 kg/m^2^)^15^. Then, the sample size was calculated using the following formula: n = [(z_1_ − α/2)^2^ × s^2^]/d^2^, with d = 2% and alpha = 0.05. The calculated sample size is 323. To strengthen the study, the obtained number was estimated to be 330. The power of the study was 80%. We used cluster random-sampling methods to recruit children. Inclusion criteria included children aged 6–12 years old, without chronic diseases such as diabetes, congenital metabolic diseases like maple syrup urine disease and phenylketonuria, thyroid gland diseases, epilepsy, and asthma, and without the use of corticosteroids, thyroid medications, diabetes medications, epilepsy medications, or allergy medications. Children were excluded if their parents did not complete the consent form. The Ethics Committee of Tehran University of Medical Sciences approved the study protocol (IR.TUMS.MEDICINE.REC.1400.582). All methods were performed in accordance with guidelines and regulations of the Ethics Committee of Tehran University of Medical Sciences. Informed consent was obtained from all participants’ legal guardians.

### Data collection tool

We used reliable and validated questionnaires, including the consent form, assessment of socioeconomic status, the International Physical Activity Questionnaire short form^16^, and a dish-based food frequency questionnaire (FFQ). The questionnaires were completed in person by asking the parents of the students.

### Dietary intake

To assess participants' dietary intake, we designed a dish-based food frequency questionnaire. The reliability and validity of questionnaire were then evaluated. The parents of the children filled out this form. The frequency of children's consumption was determined by parents based on their consumption in the last year on a daily, weekly, or monthly basis. The weights listed for each food item were converted to grams using the instructions for home scales. The NUTRITIONIST 4 program (First Data Bank, San Bruno, California) was used to assess dietary intakes to estimate energy and nutrient consumption.

### Demographic and socioeconomic status

Using a valid and reliable questionnaire created to measure SES and its association with health outcomes among Iranians, demographic and socioeconomic status (SES) were evaluated. The education and job of parents, family size, being a landlord or tenant, owning a car, number of cars, number of rooms in the house, and having appliances such as washing machine, dishwasher, LCD TV, side-by-side refrigerator, air conditioner, vacuum cleaner, computer, laptop, and advanced heating system were the items asked from parents^17^. To determine the socioeconomic status score, each questionnaire item was coded. Finally, the codes were summed and sorted into weak (1–5 scores), medium (6–10 scores), and rich (11–15 scores) categories for qualitative description.

### Anthropometric indices

The anthropometry parameters—weight, height, and mid-upper arm circumference (MUAC)—were measured for all children. A digital scale with a precision of 100 g was used to measure body weight with minimal clothing and no shoes. Height was measured using a plastic tape attached to the wall with 0.1 cm precision while standing in a normal position without shoes. MUAC was measured using a tape at the point between the shoulder and elbow (i.e., the olecranon process and the acromion). Body weight (in kilograms) was divided by the square of height (in square meters) to calculate BMI. To calculate z-scores for BMI-for-age (BAZ), height-for-age (HAZ) and weight-for-age (WAZ), the guidelines of the World Health Organization (WHO) were used. Thus, BMI for age and sex were categorized as obese (BAZ ≥ 2 SD), overweight (BAZ ≥ 1 SD), healthy weight (BAZ ≥ − 1 SD and < − 1 SD), and underweight (BAZ < − 2 SD). Categories for HAZ are (HAZ ≤ − 2 SD) short stature and (HAZ ≤ − 3 SD) severe short stature. Categories for WAZ are as follows: − 3 SD < WAZ < − 1 SD is considered underweight, and WAZ ≤ − 2 SD is considered severely underweight^18^.

### Physical activity

The International Physical Activity Questionnaire (IPAQ) short Form is used to assess physical activity (PA) as a covariate. Scores are determined based on the frequently and duration of engagement in low, moderate, high-intensity, and sedentary activities over the previous seven days. For further analysis, the proportion of daily minutes spent in moderate to vigorous physical activity (MVPA) was determined using formula outlined by Saint-Maurice et al.^19^. Thus, two categories were created: those who met physical activity guidelines (≥ 60 min per day) and those who did not meet them (< 60 min per day), based on the global recommendation of 60 min of MVPA per day (on average)^20^.

### Quality and quantity of macronutrients

The amount of proteins and fats in children's diets was calculated as a percentage of daily calories and grams per day. The quality of consumed fats was evaluated by analyzing the sources of trans fatty acids (TFAs), vegetable oils, solid vegetable oils, animal oils, cholesterol (mg/1000 kcal), omega-6 Polyunsaturated fatty acid (PUFA), omega-3 PUFA, and (PUFAs + Monounsaturated fatty acids (MUFAs))/Saturated fatty acid (SFAs), PUFAs/SFAs, and (MUFAs + PUFAs)/(SFAs + TFAs)^21,22^. Dietary protein was segregated into two categories to assess its quality: plant-based and animal-based proteins and their sources. Food/drink sources for each fat and protein intake category are shown in Supplementary Table 1.

### Validity and reliability of food frequency questionnaire

In this study, to determine the reproducibility, the questionnaire was completed by 56 parents 12 weeks apart and measured using the intra-class correlation coefficient. Three 24-h recalls were collected during the study. Pearson correlations and the Wilcoxon signed-rank test were used to assess validity. To evaluate the strength of the relation for both validity and reliability data, the following correlation rating interpretations were used: Pearson statistics—0.10 to 0.30 weak, 0.30 to 0.50 moderate, > 0.50 strong. ICC statistics—0.00 to 0.10 none, 0.11 to 0.40 slight, 0.41 to 0.60 fair, 0.61 to 0.80 moderate, and 0.81 to 1.0 substantial.

### Data analysis

The normality of variables was examined using the histogram and Kolmogorov–Smirnov test. The logarithmic equivalent (Ln transformation) was used when analyzing variables with a non-normal distribution. To evaluate the relationship between protein and fat intake as independent variables and anthropometric indices as dependent variables, one-way ANOVA was utilized. Additionally, the chi-square test was used for descriptive characteristics (i.e., supplement use, socioeconomic level, etc.) to compare the distribution of individuals across groups. An analysis of covariance was conducted to adjust for potential confounding variables: model 2 adjusted for age, supplement use, parents’ smoking, physical activity, and socioeconomic status and model 3 adjusted for age, supplement use, parents’ smoking, physical activity, socioeconomic status, and energy intake. In order to determine the odds ratios and 95% confidence intervals for the risk of underweight and overweight/obesity across tertiles of anthropometric indices in unadjusted and adjusted models, binary logistic regression was used. The regression adjusted model adjusted for age, supplement use, parents' smoking, physical activity, socioeconomic status, and energy intake. In the crude and adjusted model, the first tertile served as the reference group, and odds ratio for other tertiles were calculated. Data were presented as mean ± standard deviation (or standard error) and odds ratio and 95% confidence interval. SPSS version 22 software was used for all statistical analyses, with a p-value < 0.05 considered statistically significant.

### Ethics approval and consent to participate

The Human Ethical Committee of Tehran University of Medical Science approved the study protocol (IR.TUMS.MEDICINE.REC.1400.582). Informed consent was obtained from a parent and/or legal guardian in the study.

## Results

### Participant characteristics

Table 1 shows the sociodemographic characteristics of the participants. The mean age of the participants was 9.02 ± 1.813 years, with a mean BMI of 17.49 ± 3.93 kg/m^2^. Among the participants, 18.5% reported using dietary supplements. Additionally, 324 (98.1%) out of 330 participants failed to meet the physical activity guidelines outlined by the World Health Organization. One hundred and eighty-six participants were classified in the low socioeconomic group.

**Table 1:** Characteristics of participants (primary school girls in Kerman) in tertiles of protein and fat quantities.

Tertiles of macronutrients quantities (g)									
Variable		Tertiles of protein quantity				Tertiles of fat quantity			
		Tertile 1 ≤ 51.52 N = 110	Tertile 2 > 51.52 < 73.15 N = 110	Tertile 3 ≥ 73.15 N = 110	p value*	Tertile 1 ≤ 50.84 N = 110	Tertile 2 > 50.84 < 72.02 N = 110	Tertile 3 ≥ 72.02 N = 110	p value⃰
Quantitative variables^a^/mean ± SD									
Age (year)		8.64 (1.79)	9.03 (1.88)	9.4 (1.76)	0.007	8.75 (1.75)	8.95 (1.87)	9.37 (1.76)	0.032
Weight (kg)		28.42 (10.32)	34.43 (12.37)	38.58 (14.17)	0.0001	28.91 (10.60)	34.04 (12.26)	38.48 (14.28)	0.0001
Height (cm)		131.74 (13.21)	137.97 (13.37)	140.32 (12.75)	0.0001	132.96 (13.18)	136.58 (13.88)	140.5 (12.66)	0.0001
BMI (kg/m^2^)		15.90 (3.03)	17.55 (3.57)	19.01 (4.44)	0.0001	15.86 (3.12)	17.67 (3.47)	18.93 (4.48)	0.0001
MUAC (cm)		20.44 (2.93)	22.00 (3.55)	23.48 (3.99)	0.0001	20.46 (2.94)	22.00 (3.42)	23.46 (4.11)	0.0001
Qualitative variables^b^/N (%)									
Grade	1st	27 (8.2%)	18 (5.5%)	13 (3.9%)	0.031	23 (7.0%)	22 (6.7%)	13 (3.9%)	0.166
	2nd	22 (6.7%)	14 (4.2%)	15 (4.5%)		22 (6.7%)	14 (4.2%)	15 (4.5%)	
	3rd	20 (6.1%)	23 (7.0%)	12 (3.6%)		23 (7.0%)	16 (4.8%)	16 (4.8%)	
	4th	12 (3.6%)	17 (5.2%)	24 (7.3%)		11 (3.3%)	20 (6.1%)	22 (6.7%)	
	5th	15 (4.5%)	17 (5.2%)	25 (7.6%)		16 (4.8%)	17 (5.2%)	24 (7.3%)	
	6th	14 (4.2%)	21 (6.4%)	21 (6.4%)		15 (4.5%)	21 (6.4%)	20 (6.1%)	
Socioeconomic status	Low	80 (24.2%)	58 (17.6%)	48 (14.5%)	0.0001	78 (23.6%)	61 (18.5%)	47 (14.2%)	0.001
	Medium	30 (9.1%)	50 (15.2%)	57 (17.3%)		31 (9.4%)	47 (14.2%)	59 (17.9%)	
	High	0 (0%)	2 (0.6%)	5 (1.5%)		1 (0.3%)	2 (0.6%)	4 (1.2%)	
Supplement use	Yes	13 (3.9%)	18 (5.5%)	29 (8.8%)	0.017	9 (2.7%)	22 (6.7%)	29 (8.8%)	0.002
	No	97 (29.4%)	92 (27.9%)	81 (24.5%)		101 (30.6%)	88 (26.7%)	81 (24.5%)	
Parents' smoking	Yes	11 (3.3%)	11 (3.3%)	16 (4.8%)	0.475	9 (2.7%)	12 (3.6%)	17 (5.2%)	0.233
	No	99 (30.0%)	99 (30.0%)	94 (28.5%)		101 (30.6%)	98 (29.7%)	93 (28.2%)	
Physical activity	≥ 60 min/day	1 (0.3%)	0 (0.0%)	5 (1.5%)	0.028	1 (0.3%)	1 (0.3%)	4 (1.2%)	0.217
	< 60 min/day	109 (33%)	110 (33.3%)	105 (31.8%)		109 (33%)	109 (33%)	106 (32.1%)	
Family member	4 or less	69 (21.2%)	79 (23.9%)	80 (24.5%)	0.284	70 (21.2%)	76 (23.0%)	82 (24.8%)	0.327
	5 to 7	40 (12.1%)	29 (8.8%)	27 (8.2%)		39 (11.8%)	31 (9.4%)	26 (7.9%)	
	More than 7	1 (0.3%)	2 (0.6%)	3 (0.9%)		1 (0.3%	3 (0.9%)	2 (0.6%)	

*BMI* body mass index, *MUAC* mid-upper arm circumference. ^a^The p value reported for the quantitative variables was resulted from one-way ANOVA, and the numbers are reported as mean ± SD. ^b^The p value for the qualitative variables was calculated by the Chi-square test, and the results are based on N (%). *p value < 0.05 shows a significant level of association.

### Validity and reliability of food frequency questionnaire

The results of this study demonstrate that the developed FFQ is reliable and valid. The correlation coefficients between dietary intake estimates obtained from the FFQ and 24-HRs recalls were 0.52 for carbohydrates, 0.54 for proteins, and 0.51 for fats. The Intraclass correlation coefficients used to assess the reproducibility of the FFQ ranged from 0.54 to 0.77 (Supplementary Table 2). Furthermore, most nutrient values did not show significant differences between the FFQ and 3-day dietary records, as indicated by the Wilcoxon signed-rank test (p ≥ 0.05). Therefore, this FFQ serves as a valuable tool for assessing dietary intake in this age group.

### Dietary intake of participants

The mean consumption of energy, macronutrients, fatty acids, vitamins, minerals, and dietary groups across the three tertiles of macronutrient consumption are shown in Supplementary Table 3. All these intakes (except energy intake) were adjusted for energy intake. Intake of energy, cholesterol, MUFA, vitamins D, A, B2, B3, B5, B6, B12, and minerals including calcium, magnesium, zinc, phosphorus, iron, potassium, as well as dairy products, grains, and meat was significantly higher in the highest tertile of protein intake compared to the lowest tertile. With increasing fat intake, the intake of energy, cholesterol, PUFA, MUFA, SFA, vitamins D, B2, B5, B6, B12, and minerals including calcium, magnesium, zinc, phosphorus, potassium, as well as dairy products and meat group increased significantly.

### Anthropometric indices across tertiles of protein and fat quantity

The results are indicated based on comparing tertile 3 with tertile 1. There was a significant direct association between protein and anthropometric indices, including MUAC (23.07 ± 0.29 vs. 20.88 ± 0.29 cm; p < 0.001), BAZ (0.65 ± 0.13 vs. − 0.34 ± 0.13; p < 0.001), and HAZ (0.77 ± 0.11 vs. 0.21 ± 0.11; p = 0.001) in Model 2. Additionally, anthropometric parameters including MUAC (23.09 ± 0.29 vs. 20.79 ± 0.29 cm; p < 0.001), BAZ (0.62 ± 0.13 vs. − 0.41 ± 0.13; p < 0.001), and HAZ (0.83 ± 0.11 vs. 0.32 ± 0.11; p = 0.012) increased significantly with an increase in fat consumption in model 2. No significant association was observed between anthropometric indices and protein and fat quantity after adjusting for energy intake in model 3. The adjustment models showed no significant association between protein and fat intake with WAZ (Supplementary Table 4).

### Anthropometric indices across tertiles of protein and fat quality

Both animal and plant-based protein sources were positively associated with anthropometric indices including MUAC (Animal: 23.09 ± 0.30 vs. 20.77 ± 0.29 cm; p < 0.001, Plant: 22.78 ± 0.30 vs. 20.75 ± 0.29 cm; p < 0.001), BAZ (Animal: 0.66 ± 0.13 vs. − 0.35 ± 0.13; p < 0.001, Plant: 0.56 ± 0.13 vs. − 0.36 ± 0.13; p < 0.001) and HAZ (Animal: 0.86 ± 0.11 vs. 0.23 ± 0.11; p = 0.001, Plant: 0.67 ± 0.11 vs. 0.15 ± 0.11; p < 0.001) in model 2. No significant association was observed between protein source intake and WAZ in model 2 (Table 2). After further adjusting for energy intake, we found a significant direct association between WAZ and animal protein sources (2.38 ± 1.23 vs. − 2.87 ± 1.12; p = 0.024), and between HAZ and plant protein sources (0.48 ± 0.16 vs. 0.34 ± 0.16; p < 0.001). Similarly, a significant association was observed between the intake of various fatty acid sources, including vegetable oil, cholesterol, omega-6 PUFA, and omega-3 PUFA, with anthropometric indices (including MUAC, BAZ, and HAZ), which was maintained after adjusting for confounders for vegetable oil, cholesterol, and omega-6 PUFA (model 2). The association between omega-3 PUFA and HAZ disappeared after adjustment (model 2). The significant direct association between solid vegetable oils and MUAC (23.33 ± 3.94 vs. 20.30 ± 2.76; p < 0.001) and HAZ (0.81 ± 1.07 vs. 0.46 ± 1.15; p = 0.036), but this association was not observed after adjustment in model 2. In model 3, we observed a significant direct association between HAZ and vegetable oil (0.68 ± 0.13 vs. 0.31 ± 0.13; p = 0.038) and between omega-3 PUFA and BAZ (0.51 ± 0.15 vs. 0.07 ± 0.08; p = 0.016) and MUAC (22.60 ± 0.34 vs. 21.76 ± 0.19; p = 0.039). In the higher tertile of animal oils, MUAC (22.46 ± 3.72 vs. 22.29 ± 3.86 cm; p = 0.041) and HAZ (0.82 ± 1.14 vs. 0.38 ± 1.33; p = 0.034) were significantly higher, but this association was not detected in the adjustment models. A significant positive relationship was observed between TFAs and MUAC after adjustment in model 2 (22.57 ± 0.29 vs. 21.45 ± 0.29 cm; p = 0.025). No significant association was found between various fatty acid sources and the WAZ of subjects (Tables 3, 4, 5, 6, 7).

**Table 2:** Association between anthropometric indices and protein quality (animal and plant protein) among primary school girls in Kerman.

Tertiles of protein quality									
Variable		Tertiles of animal protein				Tertiles of plant protein			
		Tertile 1 ≤ 30.14 N = 110	Tertile 2 > 30.14 < 44.32 N = 110	Tertile 3 ≥ 44.32 N = 110	p value*	Tertile 1 ≤ 20.76 N = 109	Tertile 2 > 20.76 < 28.4 N = 111	Tertile 3 ≥ 28.4 N = 110	p value*
MUAC (cm)	Model 1^a^	20.49 (2.87)	21.91 (3.66)	23.51 (3.93)	< 0.001	20.30 (2.76)	22.27 (3.72)	23.33 (3.94)	< 0.001
	Model 2^b^	20.77 (0.29)	22.05 (0.28)	23.09 (0.30)	< 0.001	20.75 (0.29)	22.37 (0.28)	22.78 (0.30)	< 0.001
	Model 3^c^	21.25 (0.35)	22.03 (0.28)	22.63 (0.34)	0.061	21.60 (0.41)	22.38 (0.28)	21.93 (0.42)	0.174
BAZ	Model 1^a^	− 0.42 (1.34)	0.24 (1.39)	0.73 (1.40)	< 0.001	− 0.45 (1.33)	0.33 (1.45)	0.65 (1.35)	< 0.001
	Model 2^b^	− 0.35 (0.13)	0.23 (0.13)	0.66 (0.13)	< 0.001	− 0.36 (0.13)	0.34 (0.13)	0.56 (0.13)	< 0.001
	Model 3^c^	− 0.11 (0.16)	0.23 (0.12)	0.44 (0.16)	0.105	0.02 (0.19)	0.35 (0.12)	0.17 (0.19)	0.246
HAZ	Model 1^a^	0.16 (1.32)	0.69 (1.14)	0.90 (1.08)	< 0.001	0.10 (1.28)	0.92 (1.11)	0.73 (1.12)	< 0.001
	Model 2^b^	0.23 (0.11)	0.67 (0.11)	0.86 (0.11)	0.001	0.15 (0.11)	0.93 (0.11)	0.67 (0.11)	< 0.001
	Model 3^c^	0.32 (0.14)	0.67 (0.11)	0.77 (0.13)	0.107	0.34 (0.16)	0.94 (0.11)	0.48 (0.16)	< 0.001
WAZ	Model 1^a^	− 1.74 (13.58)	0.47 (1.32)	0.99 (1.28)	0.073	− 0.26 (1.22)	0.66 (1.32)	− 0.95 (14.98)	0.454
	Model 2^b^	− 1.70 (0.91)	0.38 (0.88)	1.05 (0.98)	0.107	− 0.05 (0.90)	0.68 (0.89)	− 1.25 (1.03)	0.378
	Model 3^c^	− 2.87 (1.12)	0.39 (0.87)	2.38 (1.23)	0.024	1.28 (1.29)	0.60 (0.89)	− 2.78 (1.48)	0.139

*MUAC* mid-upper arm circumference, *BAZ* BMI-for-age z-score, *HAZ* height-for-age z-score, *WAZ* weight-for-age z-score.

^a^Model 1 was resulted from one-way ANOVA, and the numbers are reported as mean ± SD. Model 1: crude.

^b^Model 2 was resulted from covariance analysis, and the numbers are reported as mean ± SE. Model 2: Adjusted for age, supplement use, parents' smoking, physical activity, and socioeconomic status.

^c^Model 3: Model 2 + energy intake.

*p value < 0.05 shows a significant level of association.

**Table 3:** Association between anthropometric indices and fat quality (vegetable oil and solid vegetable oil) among primary school girls in Kerman.

Tertiles of fat quality									
Variable		Tertiles of vegetable oil				Tertiles of solid vegetable oil			
		Tertile 1 ≤ 7.61 N = 105	Tertile 2 > 7.61 < 9.84 N = 115	Tertile 3 ≥ 9.84 N = 110	p value*	Tertile 1 ≤ 0.4 N = 129	Tertile 2 > 0.4 < 1.4 N = 76	Tertile 3 ≥ 1.4 N = 125	p value*
MUAC (cm)	Model 1^a^	20.49 (2.87)	21.91 (3.66)	23.51 (3.93)	< 0.001	20.30 (2.76)	22.27 (3.72)	23.33 (3.94)	< 0.001
	Model 2^b^	20.77 (0.29)	22.05 (0.28)	23.09 (0.30)	< 0.001	20.75 (0.29)	22.37 (0.28)	22.78 (0.30)	0.359
	Model 3^c^	21.91 (0.33)	21.85 (0.27)	22.16 (0.33)	0.785	22.13 (0.27)	21.91 (0.33)	21.85 (0.28)	0.782
BAZ	Model 1^a^	− 0.24 (1.46)	0.10 (1.29)	0.67 (1.47)	< 0.001	0.04 (1.48)	0.18 (1.48)	0.32 (1.40)	0.317
	Model 2^b^	− 0.14 (0.13)	0.12 (0.13)	0.56 (0.13)	0.002	0.01 (0.12)	0.23 (0.16)	0.32 (0.12)	0.229
	Model 3^c^	0.17 (0.15)	0.14 (0.12)	0.23 (0.15)	0.885	0.21 (0.12)	0.218 (0.15)	0.13 (0.12)	0.898
HAZ	Model 1^a^	0.17 (1.28)	0.71 (1.23)	0.86 (1.06)	< 0.001	0.46 (1.15)	0.44 (1.50)	0.81 (1.07)	0.036
	Model 2^b^	0.20 (0.11)	0.75 (0.10)	0.79 (0.11)	< 0.001	0.50 (0.10)	0.46 (0.13)	0.76 (0.10)	0.140
	Model 3^c^	0.31 (0.13)	0.75 (0.10)	0.68 (0.13)	0.038	0.60 (0.11)	0.45 (0.13)	0.66 (0.11)	0.486
WAZ	Model 1^a^	− 0.08 (1.28)	− 0.95 (13.36)	0.79 (1.39)	0.398	0.27 (1.37)	− 1.78 (16.67)	0.45 (1.26)	0.214
	Model 2^b^	− 0.064 (0.91)	− 0.94 (0.88)	0.76 (0.98)	0.443	0.20 (0.84)	− 1.84 (1.08)	0.56 (0.86)	0.196
	Model 3^c^	− 0.17 (1.06)	− 0.94 (0.88)	0.90 (1.17)	0.447	0.24 (0.88)	− 1.84 (1.08)	0.52 (0.90)	0.200

*MUAC* mid-upper arm circumference, *BAZ* BMI-for-age z-score, *HAZ* height-for-age z-score, *WAZ* weight-for-age z-score.

^a^Model 1 was resulted from one-way ANOVA, and the numbers are reported as mean ± SD. Model 1: crude.

^b^Model 2 was resulted from covariance analysis, and the numbers are reported as mean ± SE. Model 2: Adjusted for age, supplement use, parents' smoking, physical activity, and socioeconomic status.

^c^Model 3: Model 2 + energy intake.

*p value < 0.05 shows a significant level of association.

**Table 4:** Association between anthropometric indices and fat quality (animal oil and cholesterol) among primary school girls in Kerman.

Tertiles of fat quality									
Variable		Tertiles of animal oil				Tertiles of cholesterol			
		Tertile 1 ≤ 1.06 N = 85	Tertile 2 > 1.06 < 2.22 N = 131	Tertile 3 ≥ 2.22 N = 114	p value*	Tertile 1 ≤ 209.86 N = 110	Tertile 2 > 209.86 < 302.96 N = 110	Tertile 3 ≥ 302.96 N = 110	p value*
MUAC (cm)	Model 1^a^	22.29 (3.86)	21.34 (3.56)	22.46 (3.72)	0.041	20.88 (3.12)	21.84 (3.70)	23.2 (3.95)	0.0001
	Model 2^b^	22.02 (0.34)	21.80 (0.27)	22.13 (0.29)	0.724	21.12 (0.29)	21.79 (0.28)	23.00 (0.29)	0.0001
	Model 3^c^	22.45 (0.33)	21.92 (0.26)	21.86 (0.29)	0.234	21.72 (0.33)	21.82 (0.28)	22.38 (0.34)	0.398
BAZ	Model 1^a^	0.16 (1.53)	0.09 (1.47)	0.30 (1.37)	0.519	− 0.23 (1.44)	0.08 (1.42)	0.69 (1.34)	0.0001
	Model 2^b^	0.15 (0.15)	0.13 (0.12)	0.26 (0.13)	0.783	− 0.15 (0.13)	0.06 (0.13)	0.64 (0.13)	0.0001
	Model 3^c^	0.34 (0.15)	0.18 (0.12)	0.05 (0.13)	0.387	0.13 (0.15)	0.07 (0.13)	0.33 (0.15)	0.438
HAZ	Model 1^a^	0.38 (1.33)	0.52 (1.19)	0.82 (1.14)	0.034	0.18 (1.3)	0.66 (1.2)	0.92 (1.05)	0.0001
	Model 2^b^	0.48 (0.13)	0.51 (0.10)	0.76 (0.11)	0.183	0.24 (0.11)	0.67 (0.11)	0.85 (0.11)	0.001
	Model 3^c^	0.57 (0.13)	0.53 (0.10)	0.66 (0.11)	0.729	0.33 (0.13)	0.67 (0.11)	0.75 (0.13)	0.100
WAZ	Model 1^a^	− 1.93 (16.52)	0.35 (1.26)	0.51 (1.28)	0.154	− 1.52 (13.53)	0.31 (1.35)	0.90 (1.24)	0.131
	Model 2^b^	− 1.74 (1.09)	0.25 (0.79)	0.51 (0.93)	0.247	− 1.37 (0.90)	0.27 (0.93)	0.78 (0.93)	0.227
	Model 3^c^	− 1.80 (1.13)	0.24 (0.79)	0.56 (0.96)	0.247	− 2.11 (1.07)	0.213 (0.93)	1.64 (1.15)	0.109

*MUAC* mid-upper arm circumference, *BAZ* BMI-for-age z-score, *HAZ* height-for-age z-score, *WAZ* weight-for-age z-score.

^a^Model 1 was resulted from one-way ANOVA, and the numbers are reported as mean ± SD. Model 1: crude.

^b^Model 2 was resulted from covariance analysis, and the numbers are reported as mean ± SE. Model 2: Adjusted for age, supplement use, parents' smoking, physical activity, and socioeconomic status.

^c^Model 3: Model 2 + energy intake.

*p value < 0.05 shows a significant level of association.

**Table 5:** Association between anthropometric indices and fat quality (omega-6 PUFAs and omega-3 PUFAs) among primary school girls in Kerman.

Tertiles of fat quality								
Variable		Tertiles of omega-6 PUFAs				Tertiles of omega-3 PUFAs		
		Tertile 1 ≤ 9.55 N = 110	Tertile 2 > 9.55 < 12.88 N = 110	Tertile 3 ≥ 12.88 N = 110	p value*	Equal or less than median ≥ 0.019 N = 247	More than median < 0.019 N = 83	p value*
MUAC (cm)	Model 1^a^	20.82 (3.09)	21.76 (3.46)	23.33 (4.12)	0.0001	21.51 (3.45)	23.34 (4.14)	0.0001
	Model 2^b^	21.24 (0.29)	21.86 (0.28)	22.82 (0.29)	0.001	21.62 (0.19)	23.01 (0.34)	0.001
	Model 3^c^	22.29 (0.38)	21.90 (0.28)	21.72 (0.39)	0.661	21.76 (0.19)	22.60 (0.34)	0.039
BAZ	Model 1^a^	− 0.33 (1.41)	0.23 (1.38)	0.65 (1.40)	0.0001	− 0.02 (1.42)	0.80 (1.35)	0.0001
	Model 2^b^	− 0.23 (0.13)	0.23 (0.13)	0.55 (0.13)	0.0001	0.01 (0.08)	0.70 (0.15)	0.0001
	Model 3^c^	0.19 (0.17)	0.24 (0.12)	0.10 (0.18)	0.804	0.07 (0.08)	0.51 (0.15)	0.016
HAZ	Model 1^a^	0.32 (1.36)	0.62 (1.14)	0.82 (1.11)	0.009	0.48 (1.21)	0.91 (1.20)	0.006
	Model 2^b^	0.35 (0.11)	0.63 (0.11)	0.78 (0.11)	0.037	0.51 (0.07)	0.81 (0.13)	0.055
	Model 3^c^	0.60 (0.15)	0.64 (0.11)	0.52 (0.15)	0.794	0.55 (0.07)	0.71 (0.13)	0.327
WAZ	Model 1^a^	− 0.04 (1.33)	− 1.10 (13.99)	0.81 (1.38)	0.336	− 0.51 (9.31)	0.99 (1.34)	0.208
	Model 2^b^	0.145 (0.87)	− 1.41 (0.92)	0.91 (0.97)	0.212	− 0.44 (0.61)	0.77 (0.77)	0.339
	Model 3^c^	− 0.08 (1.21)	− 1.40 (0.92)	1.17 (1.37)	0.211	− 0.44 (0.62)	0.79 (1.13)	0.354

*MUAC* mid-upper arm circumference, *BAZ* BMI-for-age z-score, *HAZ* height-for-age z-score, *WAZ* weight-for-age z-score, *PUFA* polyunsaturated fatty acid.

^a^Model 1 was resulted from one-way ANOVA, and the numbers are reported as mean ± SD. Model 1: crude.

^b^Model 2 was resulted from covariance analysis, and the numbers are reported as mean ± SE. Model 2: Adjusted for age, supplement use, parents' smoking, physical activity, and socioeconomic status.

^c^Model 3: Model 2 + energy intake.

*p value < 0.05 shows a significant level of association.

**Table 6:** Association between anthropometric indices and fat quality (trans fatty acids and PUFAs/SFAs) among primary school girls in Kerman.

Tertiles of fat quality									
Variable		Tertiles of TFAs				Tertiles of PUFAs/SFAs			
		Tertile 1 ≤ 0.41 N = 108	Tertile 2 > 0.41 < 0.71 N = 111	Tertile 3 ≥ 0.71 N = 111	p value*	Tertile 1 ≤ 1.46 N = 103	Tertile 2 > 1.46 < 1.65 N = 115	Tertile 3 ≥ 1.65 N = 112	p value*
MUAC (cm)	Model 1^a^	21.47 (3.21)	21.91 (3.71)	22.52 (4.13)	0.111	22.42 (3.47)	21.58 (3.41)	21.96 (4.20)	0.247
	Model 2^b^	21.45 (0.29)	21.88 (0.29)	22.57 (0.29)	0.025	22.32 (0.30)	21.82 (0.29)	21.80 (0.29)	0.384
	Model 3^c^	21.95 (0.30)	21.80 (0.28)	22.16 (0.29)	0.680	22.26 (0.29)	21.86 (0.28)	21.82 (0.28)	0.495
BAZ	Model 1^a^	− 0.00 (1.40)	0.15 (1.51)	0.39 (1.43)	0.115	0.32 (1.38)	0.22 (1.40)	0.01 (1.56)	0.293
	Model 2^b^	0.01 (0.13)	0.11 (0.13)	0.41 (0.13)	0.100	0.30 (0.13)	0.25 (0.13)	0.00 (0.13)	0.240
	Model 3^c^	0.26 (0.13)	0.08 (0.12)	0.21 (0.13)	0.618	0.276 (0.13)	0.274 (0.12)	0.007 (0.12)	0.247
HAZ	Model 1^a^	0.41 (1.40)	0.61 (1.12)	0.74 (1.12)	0.127	0.68 (1.12)	0.63 (1.23)	0.46 (1.31)	0.377
	Model 2^b^	0.44 (0.11)	0.60 (0.11)	0.72 (0.11)	0.212	0.64 (0.11)	0.61 (0.11)	0.52 (0.11)	0.736
	Model 3^c^	0.56 (0.12)	0.58 (0.11)	0.62 (0.11)	0.945	0.62 (0.11)	0.62 (0.11)	0.52 (0.11)	0.770
WAZ	Model 1^a^	0.10 (1.31)	0.35 (1.34)	− 0.82 (13.61)	0.610	0.55 (1.24)	0.51 (1.37)	− 1.51 (14.05)	0.182
	Model 2^b^	0.19 (0.94)	0.21 (0.91)	− 0.77 (0.89)	0.676	0.43 (0.95)	0.60 (0.88)	− 1.50 (0.93)	0.216
	Model 3^c^	0.41 (1.01)	0.18 (0.91)	− 0.94 (0.93)	0.583	0.42 (0.96)	0.59 (0.88)	− 1.50 (0.93)	0.219

*MUAC* mid-upper arm circumference, *BAZ* BMI-for-age z-score, *HAZ* height-for-age z-score, *WAZ* weight-for-age z-score, *PUFA* polyunsaturated fatty acid, *SFA* saturated fatty acid, *TFA* trans fatty acid.

^a^Model 1 was resulted from one-way ANOVA, and the numbers are reported as mean ± SD. Model 1: crude.

^b^Model 2 was resulted from covariance analysis, and the numbers are reported as mean ± SE. Model 2: Adjusted for age, supplement use, parents' smoking, physical activity, and socioeconomic status.

^c^Model 3: Model 2 + energy intake.

*p value < 0.05 shows a significant level of association.

**Table 7:** Association between anthropometric indices and fat quality (PUFAs + MUFAs/SFAs and (MUFAs + PUFAs)/(SFAs + TFAs)) among primary school girls in Kerman.

Tertiles of fat quality									
Variable		Tertiles of PUFAs + MUFAs/SFAs				Tertiles of (MUFAs + PUFAs)/(SFAs + TFAs)			
		Tertile 1 ≤ 0.59 N = 103	Tertile 2 > 0.59 < 0.7 N = 115	Tertile 3 ≥ 0.7 N = 112	p value*	Tertile 1 ≤ 1.42 N = 99	Tertile 2 > 1.42 < 1.6 N = 117	Tertile 3 ≥ 1.6 N = 114	p value*
MUAC (cm)	Model 1^a^	22.37 (3.65)	21.61 (3.17)	21.97 (4.26)	0.322	22.31 (3.59)	21.76 (3.29)	21.90 (4.22)	0.538
	Model 2^b^	22.31 (0.30)	21.75 (0.28)	21.89 (0.29)	0.390	22.20 (0.31)	21.89 (0.28)	21.86 (0.29)	0.686
	Model 3^c^	22.26 (0.29)	21.81 (0.27)	21.87 (0.28)	0.481	22.17 (0.29)	21.92 (0.27)	21.85 (0.28)	0.715
BAZ	Model 1^a^	0.36 (1.51)	0.10 (1.27)	0.09 (1.57)	0.306	0.27 (1.43)	0.21 (1.32)	0.07 (1.60)	0.569
	Model 2^b^	0.34 (0.13)	0.12 (0.13)	0.09 (0.13)	0.372	0.25 (0.14)	0.23 (0.13)	0.07 (0.13)	0.575
	Model 3^c^	0.32 (0.13)	0.14 (0.12)	0.08 (0.12)	0.422	0.24 (0.13)	0.24 (0.12)	0.06 (0.12)	0.526
HAZ	Model 1^a^	0.67 (1.23)	0.61 (1.17)	0.49 (1.28)	0.554	0.62 (1.25)	0.63 (1.12)	0.51 (1.30)	0.704
	Model 2^b^	0.61 (0.11)	0.58 (0.11)	0.57 (0.11)	0.961	0.58 (0.12)	0.60 (0.11)	0.58 (0.11)	0.991
	Model 3^c^	0.60 (0.11)	0.59 (0.11)	0.56 (0.11)	0.970	0.57 (0.11)	0.61 (0.11)	0.58 (0.11)	0.974
WAZ	Model 1^a^	0.64 (1.32)	0.37 (1.30)	− 1.44 (14.05)	0.209	0.49 (1.25)	0.52 (1.34)	− 1.40 (13.80)	0.221
	Model 2^b^	0.49 (0.94)	0.47 (0.88)	− 1.42 (0.93)	0.260	0.37 (0.98)	0.61 (0.87)	− 1.40 (0.91)	0.245
	Model 3^c^	0.49 (0.95)	0.47 (0.89)	− 1.42 (0.94)	0.261	0.37 (0.98)	0.61 (0.87)	− 1.39 (0.92)	0.249

*MUAC* mid-upper arm circumference, *BAZ* BMI-for-age z-score, *HAZ* height-for-age z-score, *WAZ* weight-for-age z-score, *PUFA* polyunsaturated fatty acid, *MUFA* monounsaturated fatty acid, *SFA* saturated fatty acid, *TFA* trans fatty acids.

^a^Model 1 was resulted from one-way ANOVA, and the numbers are reported as mean ± SD. Model 1: crude.

^b^Model 2 was resulted from covariance analysis, and the numbers are reported as mean ± SE. Model 2: Adjusted for age, supplement use, parents' smoking, physical activity, and socioeconomic status.

^c^Model 3: Model 2 + energy intake.

*p value < 0.05 shows a significant level of association.

### Odds ratio and 95% confidence interval for weight disorders in tertiles of protein and fat quantity

**Figure 1:**
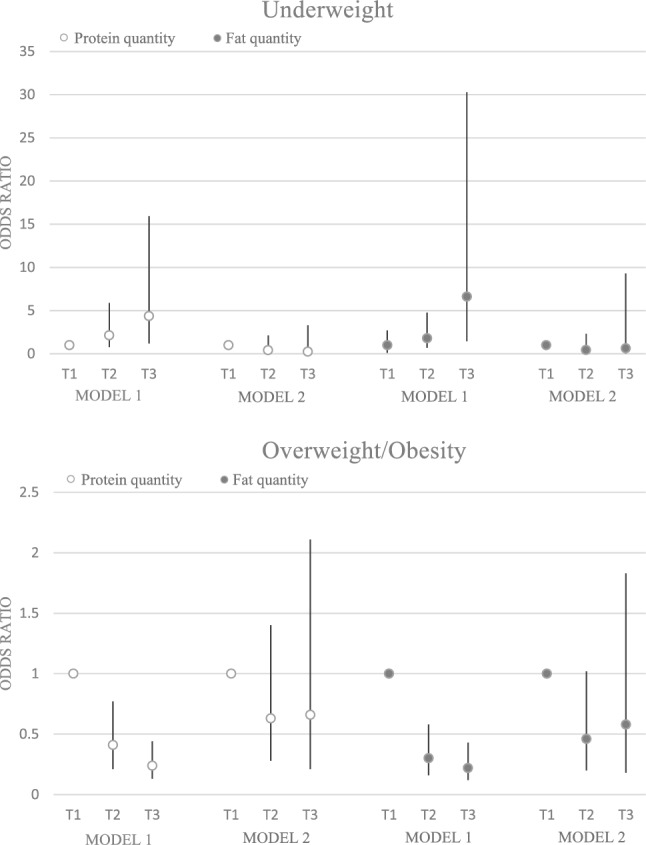
Odds ratio and 95% confidence interval for weight disorders in tertile of protein and fat quantities. Model 1: crude. Model 2: Adjusted for age, supplement use, parents' smoking, physical activity, socioeconomic status, and energy intake.

Figure 1 illustrates the odds ratio and 95% confidence interval for underweight, overweight, and obesity in tertiles of protein and fat quantity. A significant association was observed between protein (OR: 0.24; 95% CI: 0.13–0.44; p trend: < 0.001) and fat intake (OR: 0.22; 95% CI: 0.12–0.43; p trend: < 0.001) with overweight and obesity in the crude model. Similarly, an association was seen between protein (OR: 4.36; 95% CI: 1.19–15.93; p trend: 0.017) and fat intake (OR: 6.61; 95% CI: 1.44–30.28; p trend: 0.009) and underweight before adjusting for confounders. However, after adjustment, no significant association was found between underweight and overweight/obesity and protein and fat intake.

### Odds ratio and 95% confidence interval for weight disorders in tertiles of protein and fat quality

**Figure 2:**
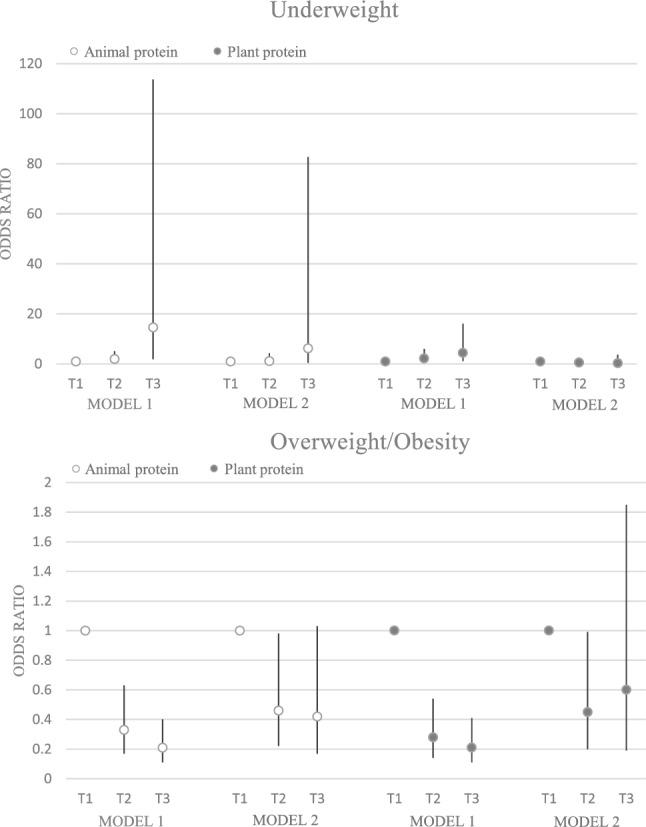
Odds ratio and 95% confidence interval for weight disorders in tertile of protein quality. Model 1: crude. Model 2: Adjusted for age, supplement use, parents' smoking, physical activity, socioeconomic status, and energy intake.

A significant association was observed between animal-based protein and underweight (OR: 14.6; 95% CI: 1.87–113.73; p trend: 0.002), and overweight/obesity (OR: 0.21; 95% CI: 0.11–0.40; p trend: < 0.001). This association did not remain significant after adjustment for confounders. There was a significant positive association between plant-based protein and underweight (OR: 4.41; 95% CI: 1.20–16.10; p trend: 0.016) and overweight/obesity (OR: 021; 95% CI: 0.11–0.41; p trend: < 0.001) before adjustment; however, no significant association was observed after adjustment between plant protein and weight disorders (including underweight, overweight and obesity) (p trend ≥ 0.05) (Fig. 2).

**Figure 3:**
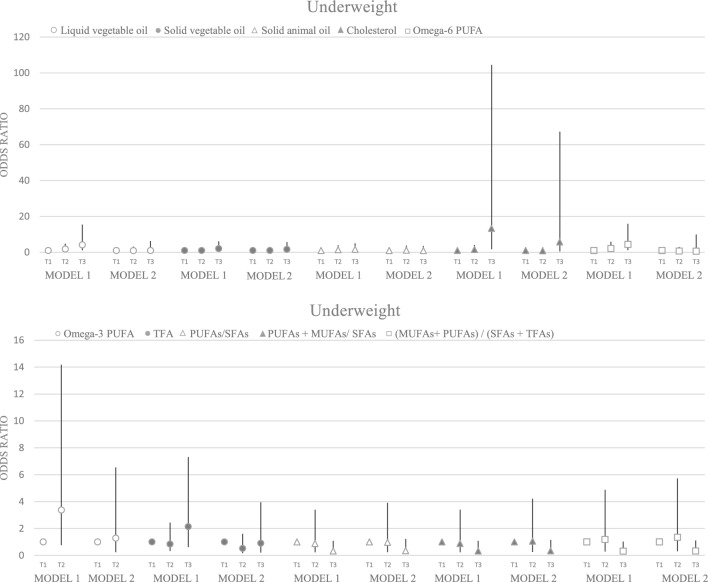
Odds ratio and 95% confidence interval for underweight in tertile of fat quality. Model 1: crude. Model 2: Adjusted for age, supplement use, parents' smoking, physical activity, socioeconomic status, and energy intake.

**Figure 4:**
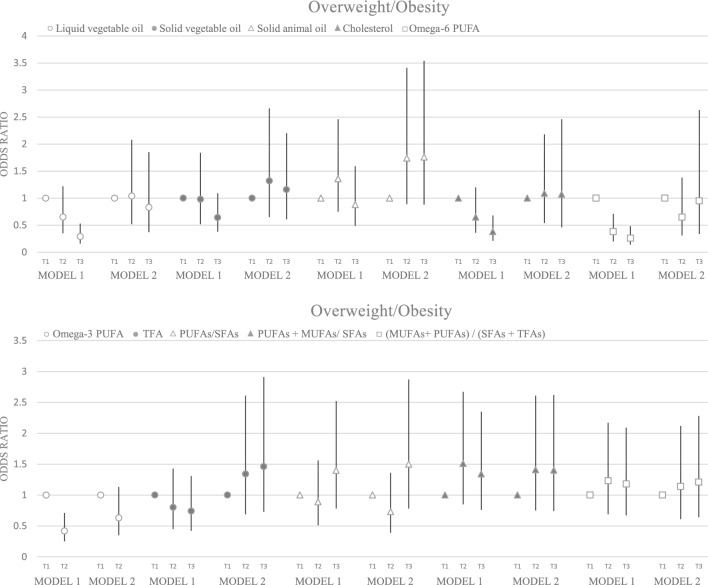
Odds ratio and 95% confidence interval for overweight/obesity in tertile of fat quality. Model 1: crude. Model 2: Adjusted for age, supplement use, parents' smoking, physical activity, socioeconomic status, and energy intake.

Significant associations were found between underweight and vegetable oil, cholesterol, omega-6, PUFAs/SFAs, (PUFAs + MUFAs)/SFAs, and (MUFAs + PUFAs)/(SFAs + TFAs) (p < 0.05), Which remained significant for (MUFAs + PUFAs)/(SFAs + TFAs) after adjustment (p = 0.040) (Fig. 3). Additionally, we found significant associations between vegetable oil (p trend < 0.001), cholesterol (p trend = 0.001), omega-6 PUFA (p trend < 0.001) and omega-3 PUFA (p trend = 0.001) and overweight in the crude model, which disappeared after adjusting for confounding variables (p trend ≥ 0.05) (Fig. 4).

## Discussion

To the best of our knowledge, this study is the first to investigate the association between the quality and quantity of protein and fat consumption with selected anthropometric indices among primary school girls in Kerman. The findings showed a positive association between animal protein intake and WAZ, plant protein intake and HAZ, and vegetable oils intake and HAZ. Moreover, a significant positive association was found between the intake of omega-3 PUFA and MUAC and BAZ. Furthermore, the risk of underweight decreased with an increase in the ratio of (MUFAs + PUFAs) to (SFAs + TFAs).

Our study found a positive association between protein intake and anthropometric indices, indicating that higher protein intake is associated with increased body size and weight. Most studies that investigated the relationship between protein quantity and anthropometric indicators had similar results to our study. Among these studies, we can mention the study by Koletzko et al.^23^ and Markides et al.^24^. However, this association disappeared when further adjusted for energy intake in model 3. This suggests that the observed relationship in model 2 may be confounded by energy intake. It is possible that individuals with higher protein intake also consume more energy, leading to increased body size and weight. In a research evaluating the effects of a high-protein diet with energy restriction on weight loss and hunger in overweight and obese children, it was found that a high-protein diet had no more significant effect on weight loss, body composition, or changes in appetite or mood compared to a standard diet^25^. The main difference between our study and this intervention is that their population is overweight and obese children, which can affect the results.

Consistent with previous findings, in the model 2 of the present study, we found a positive relationship between animal protein intake and anthropometric indices (MUAC, BAZ, and HAZ). Studies have demonstrated the positive effect of high-quality protein on children's height^26,27^. In model 2, we did not find a significant association between animal protein intake and WAZ, but in model 3, which further adjusted for energy intake, a significant association was observed. In a cross-sectional study, Dor et al. found that high daily protein intake, mainly from non-dairy animal sources, was positively associated with overweight/obesity in adolescents^28^. Conversely, a study on adults indicated that animal protein intake was associated with lower odds of overweight/obesity^29^.

The positive association between animal protein and WAZ may be attributed to the idea that protein, particularly from animal sources, could contribute to the accumulation of body fat by increasing levels of insulin-like growth factor-1 (GLP-1)^28^.

The findings of our study suggest that plant protein intake may have a positive association with MUAC, BAZ, and HAZ in children after adjusting for potential confounding factors such as age, supplement use, parents’ smoking, physical activity, and socioeconomic status. Importantly, the significant association with HAZ remained even after further adjusting for energy intake, indicating that plant protein intake may have a direct effect on linear growth independent of total calorie consumption. These results highlight the importance of considering both the quantity and quality of protein sources in children’s diets, and suggest that increasing plant protein intake may be beneficial for promoting healthy growth and development.

The results of our study suggest that there is a positive association between amount of fat intake and anthropometric indices, including MUAC, BAZ, and HAZ, when adjusting for age, supplement use, parents’ smoking, physical activity, and socioeconomic status. This finding is consistent with previous research that has also reported a positive association between fat intake and overweight^30^. However, when we further adjusted for energy intake in model 3, the association between fat intake and anthropometric indices became non-significant. This suggests that the observed positive association in model 2 may be largely by the energy content of the diet. A cross-sectional study from Ghana reported an inverse association between the percentage of energy obtained from total fat and overweight/obesity^31^.

Nevertheless, calories from fat alone may have little effect on growth. It seems that the type of fatty acid is more important^32^. The present study found a positive relationship between HAZ and intake of vegetable oils. Vegetable oils are rich in essential fatty acids, such as omega-3 and omega-6, which play a crucial role in growth and development. A cross-sectional study found a significant positive association between omega-6 PUFA and height Z-score in children^33^. Furthermore, vegetable oils contain fat-soluble vitamins, including vitamin E and vitamin D, which are essential for bone health and may contribute to the growth of children^34–38^.

According to our findings, a higher intake of omega-3 was associated with higher MUAC and BAZ. Consistent with our study, several studies have shown a positive relationship between omega-3 intake and weight. A systematic review and meta-analysis did not show a statistically significant effect of omega-3 on lean body mass and BMI but did show a significant effect on body weight^39^. Additionally, the Nurses’ Health Study found that women who consumed more fish or servings of fish per week were at a greater risk of being overweight^40^.

On the other hand, some studies have reported conflicting results. In a study on 134 normal-weight and overweight children, obese children had a negative association between their BMI Z-score and plasma n-3 PUFA and DHA, despite having higher intakes of PUFA^41^. Another study examining the dietary intake of 132 4-year-old children reported that low omega-3 PUFA intake was associated with higher body weight^42^. Some studies found no correlation between omega-3 intake and weight changes^43,44^.

The findings of our study indicate that there is a significant negative association between the ratio of (MUFAs + PUFAs)/(SFAs + TFAs) and the risk of underweight, even after adjusting for various confounding variables. There is no study that has reported the association between (MUFAs + PUFAs)/(SFAs + TFAs) and risk of underweight. Most of the research on this topic focuses on the relationship between this ratio and overweight and obesity. It is possible that a higher intake of unsaturated fats may provide essential nutrients and promote healthy weight gain. Future research should explore the potential biological mechanisms and determine if this association persists over time.

In addition to protein and fat, carbohydrates as another macronutrient have an important effect on anthropometric indices. In continuation of our research on the dietary factors influencing anthropometric indices among primary school girls in Kerman city, we conducted a study on the association of quality and quantity of carbohydrate intake with selected anthropometric indices. In this study, we found positive associations between carbohydrate quantity, glycemic load, and fiber intake with MUAC, BAZ, and HAZ, as well as an inverse association between BAZ and the ratio of solid carbohydrates to total carbohydrates^45^.

### Strengths and limitations

The present study has important strengths. One of its strengths was that our study was the first to examine the connection between the type and quantity of protein and fat and the status of weight and height in primary school girls in Kerman. As a result, programs focused on preventing and reducing weight and height issues in this age group can benefit from our study's results on the quality and quantity of protein and fat consumed by primary school girls. The validity and reliability of the 185-item food frequency questionnaire were assessed in this study, which was mainly created for the city of Kerman and included most of the local food consumed by our study population. The impact of several potential confounders, such as age, parents' education, parents' occupation, supplement use, parents' smoking, physical activity, socioeconomic status, and energy intake has been considered in this study. Furthermore, for eight weeks, parents of children received instruction on healthy eating, and the efficacy of this instruction was examined through a study effort. Therefore, by teaching and informing parents, our work was valuable and applicable to society.

However, this research has several limitations. A definitive cause-and-effect association between protein and fat quality and quantity and weight and height status cannot be established due to the study's cross-sectional approach. Another drawback of our study was the use of the FFQ to measure protein and fat consumption, which may underestimate or overestimate absolute quantities of intake. Lastly, we used the IPAQ-short form questionnaire, which is commonly used in adults and adolescents older than 16 years old, to assess physical activity in primary school girls. Thus, it is recommended to use a questionnaire particularly developed for children in future studies. Furthermore, it is recommended that future research explore the association between the quality and quantity of protein and fat intake and the status of height and weight in primary school pupils prospectively. Due to different food habits, further studies should also be conducted in other age and population groups.

## Conclusion

In conclusion, the present study suggests significant associations between protein and fat intake and various anthropometric indices among primary school girls. Plant proteins and vegetable oils intake was positively associated with height, while animal protein intake was linked to weight. Additionally, omega-3 PUFA were positively associated with MUAC and BAZ. Further research is required to confirm these associations.

### Supplementary Information

Supplementary Tables.

## Data Availability

The data sets used and analyzed during the current study are available from the corresponding author upon reasonable request.
