# Supplementary material for: Protein and fat intake impact on growth of primary school girls in Kerman, Iran
Source: Sci Rep. 2024 Jul 3;14:15288. doi: 10.1038/s41598-024-66001-4 (PMC11222480; doi:10.1038/s41598-024-66001-4)
Supplement: Supplementary file 1 — Supplementary Tables. [file 41598_2024_66001_MOESM1_ESM.docx]

**Protein and fat intake impact on growth of primary school girls in Kerman**

**Nooshin Jannati^1^, Mohammad Reza Mahmoodi^2**^, Leila** **Azadbakht^1*^**

^1^Department of Community Nutrition, School of Nutritional Sciences and Dietetics, Tehran University of Medical Sciences (TUMS), Tehran, Iran.

^2^Physiology Research Center, Institute of Neuropharmacology & Department of Nutrition, Faculty of Public Health, Kerman University of Medical Sciences, Kerman, Iran.

*******Corresponding author:**

Leila Azadbakht

azadbakhtleila@gmail.com

****Co-corresponding author:**

Mohammad Reza Mahmoodi

mahmoodimr@yahoo.com

**Supplementary table 1.** Food/drink sources for fat and protein intake category

| **Animal protein** | **Animal oil** | **TFA** |
| --- | --- | --- |
| Low/medium/ high fat milk | Butter | Cake |
| Mixed milk | Animal oil | Biscuit |
| Low/medium/high fat yogurt |  | Cookie |
| Creamy yogurt |  | Popcorn |
| Cheese |  | Chips |
| Creamy cheese |  | Cheese puffs |
| Cream |  | Sausage |
| Butter |  | Bologna |
| Dough |  | French fries |
| Pasteurized/traditional curd |  | Mayonnaise sauce |
| Pasteurized/traditional ice cream |  | Cream |
| Egg |  | Creamy cheese |
| Chicken |  | Butter |
| Sausage |  | Animal oil |
| Bologna |  | Vegetable oil |
| Abgoosht |  | Solid vegetable oil |
| Meat (lamb, beef,.( |  |  |
| Brain |  |  |
| Sirabi |  |  |
| Kale Pache |  |  |
| Liver |  |  |
| Fish |  |  |
| Canned fish |  |  |

Plant protein: All protein except animal protein

**Supplementary table 2.** Reproducibility and validation study: Correlation coefficient and Wilcoxon signed-rank test for validity and ICC for reproducibility.

|  | *FFQ* | *3DR* | *Wilcoxon Signed Rank Test (p Value)^a^* | *Correlation coefficient^b^* | *ICC^c^* |  |
| --- | --- | --- | --- | --- | --- | --- |
|  | *Mean ± SD* | *Mean ± SD* |  |  |  |  |
| *Carbohydrate*  *Protein*  *Fat*  *Calcium*  *Fe*  *Magnesium*  *Vitamin C*  *Vitamin A*  *Vegetables*  *Fruits*  *Meat and its products*  *Dairy products* | | 262.22(92.28) | 235.75(84.97) | 0.013 | 0.7  0.63  0.61  0.54  0.75  0.65  0.77  0.68  0.59  0.68  0.68  0.89 | 0.52  0.54  0.51  0.59  0.74  0.60  0.62  0.70  0.75  0.65  0.75  0.62 |
|  |  | 63.42(22.62) | 58.75(20.94) | 0.059 |  |  |
|  |  | 64.02(25.76) | 60.28(24.58) | 0.136 |  |  |
|  |  | 748.58(335.10) | 696.48(272.37) | 0.434 |  |  |
|  |  | 13.65(5.01) | 12.77(4.78) | 0.016 |  |  |
|  |  | 218.52(76.82) | 201.07(81.82) | 0.013 |  |  |
|  |  | 97.48(54.55) | 95.75(62.34) | 0.211 |  |  |
|  |  | 777.25(503.65) | 745.43(532.87) | 0.915 |  |  |
|  |  | 170.65(87.34) | 175.79(107.75) | 0.866 |  |  |
|  |  | 280.38(155.49) | 284.13(170.27) | 0.135 |  |  |
|  |  | 89.56(41.06) | 78.41(34.25) | 0.124 |  |  |
|  |  | 394.29(224.29) | 370.29(192.16) | 0.541 |  |  |

^a^Wilcoxon signed-rank test were used to examine the difference between FFQ and 3DR with a significant p value<0.001.

^b^Pearson or Spearman correlation coefficient were used to assess the correlation between variables with a p-value < 0.05 considered as significant.

^c^the intra-class coefficient for the comparison between FFQ1 and FFQ2. 3DR, dietary recall.

**Supplementary table 3.** Dietary intake of participants (primary school girls in Kerman) in tertiles of protein and fat quantity.

| Tertiles of macronutrient quantity | | | | | | | | |
| --- | --- | --- | --- | --- | --- | --- | --- | --- |
| Tertiles of fat quantity | | | | Tertiles of protein quantity | | | |  |
| p value* | Tertile 3  72.02≤  N= 110 | Tertile 2  >50.84 <72.02  N= 110 | Tertile 1  ≤50.84  N= 110 | p value* | Tertile 3  73.15≤  N= 110 | Tertile 2  >51.52 <73.15  N= 110 | Tertile 1  ≤51.52  N= 110 | Variable |
| <0.001  0.909  <0.001  0.011  <0.001  0.045 | 2513.64 (474.28)  14.01 (0.35)  297.65 (8.90)  24.03 (0.90)  22.37 (0.04)  14.92 (0.42) | 1814.85 (226.68)  14.2 (0.23)  266.77 (5.94)  20.85 (0.60)  19.01 (0.28)  13.63 (0.28) | 1206.23 (290.28)  14.23 (0.34)  220.46 (8.68)  19.74 (0.87)  16.79 (0.41)  13.35 (0.41) | <0.001  0.195  <0.001  0.445  0.003  0.988 | 2520.85 (468.09)  13.77 (0.365)  312.91 (8.93)  22.46 (0.93)  20.80 (0.46)  13.92 (0.44) | 1817.05 (212.34)  14.48 (0.238)  259.55 (5.82)  21.04 (0.60)  19.32 (0.30)  14.00 (0.28) | 1196.81 (272.93)  14.19 (0.357)  212.43 (8.72(  21.13 (0.91)  18.04 (0.45)  13.98 (0.43) | Energy (kcal/d)  fiber (g/d)  Cholesterol (mg/d)  SFA (g/d)  MUFA (g/d)  PUFA (g/d) |
| 0.984  0.001  0.391  0.854  0.082  <0.001  0.801  0.001  <0.001  0.587  <0.001  0.745 | 734.62 (65.67)  1.84 (0.14)  11.35 (0.40)  164.66 (6.62)  1.55 (0.03)  1.67 (0.03)  16.24 (0.27)  5.41 (0.10)  1.69 (0.05)  217.64 (6.12)  3.97 (0.11)  93.20 (6.03) | 748.88 (43.82)  1.48 (0.09)  11.59 (2.73)  168.14 (4.42)  1.63 (0.02)  1.49 (0.02)  16.27 (0.18)  4.99 (0.07)  1.51 (0.03)  214.76 (4.08)  3.28 (0.07)  98.33 (4.02) | 752.78 (64.05)  0.945 (0.14)  12.22 (0.39)  165.55 (6.46)  1.68 (0.03)  1.29 (0.03)  16.06 (0.26)  4.74 (0.10)  1.27 (0.05)  207.79 (5.97)  2.69 (0.10)  100.89 (5.88) | 0.039  <0.001  0.222  0.755  0.455  <0.001  0.001  0.004  0.026  0.618  <0.001  0.535 | 815.73 (66.6)  1.97 (0.14)  11.05 (0.41)  167.01 (6.78)  1.59 (0.036)  1.73 (0.03)  17.13 (0.27)  5.38 (0.10)  1.53 (0.05)  215.28 (6.27)  4.21 (0.10)  91.08 (6.17) | 808.43 (43.39)  1.42 (0.09)  11.84 (0.27)  168.45 (4.41)  1.64 (0.02)  1.47 (0.02)  16.14 (0.18)  5.01 (0.07)  1.55 (0.03)  215.95 (4.08)  3.14 (0.07)  99.01 (4.02) | 612.13 (65.03)  0.89 (0.14)  12.28 (0.40)  162.88 (6.62)  1.63 (0.035)  1.25 (0.03)  15.3 (0.26)  4.75 (0.10)  1.39 (0.05)  208.95 (6.12)  2.59 (0.10)  102.32 (6.02) | Vitamin A (RAE)  Vitamin D (μg)  Vitamin E (mg)  Vitamin K (mg)  Thiamine (mg)  Riboflavin (mg)  Niacin (mg)  Vitamin B5 (mg)  Vitamin B6 (mg)  Folic acid (μg)  Vitamin B12 (μg)  Vitamin C (mg) |
| 0.001  0.001  <0.001  <0.001  0.377  <0.001  0.406 | 845.71 (29.92)  231.43 (3.99)  3010.86 (58.54)  9.02 (0.15)  13.82 (0.27)  1200.01 (22.96)  0.10 (0.003) | 748.11 (19.96)  218.2 (2.66)  2819.28 (39.06)  8.06 (0.10)  13.79 (0.18)  1066.73 (15.32)  0.099 (0.002) | 651.91 (29.18)  205.92 (3.89)  2591.93 (57.09)  7.26 (0.15)  13.35 (0.26)  949.8 (22.39)  0.103 (0.003) | <0.001  0.001  <0.001  <0.001  0.002  <0.001  0.753 | 870.00 (30.39)  231.7 (4.08)  2997.75 (60.09)  9.15 (0.16)  14.46 (0.27)  1176.83 (23.99)  0.10 (0.003) | 740.05 (19.8)  218.71 (2.66)  2831.55 (39.14)  7.98 (0.10)  13.71 (0.17)  1072.6 (15.63)  0.100 (0.002) | 635.67 (29.68)  205.14 (3.98)  2592.77 (58.67)  7.21 (0.15)  12.79 (0.26)  967.21 (23.42)  0.103 (0.003) | Calcium (mg)  Magnesium (mg)  Potassium (mg)  Zinc (mg)  Fe (mg)  Phosphorus (mg)  Selenium (mg) |

SFA= saturated fatty acids, MUFA= monounsaturated fatty acids, and PUFA= polyunsaturated fatty acids. The p value is reported from covariance analysis, and the results are based on mean ± SD. All of the variables are adjusted for energy intake. *p value <0.05 shows a significant level of association

**Supplementary table 3.** Continued.

| Tertiles of macronutrient quantity | | | | | | | | |
| --- | --- | --- | --- | --- | --- | --- | --- | --- |
| Tertiles of fat quantity | | | | Tertiles of protein quantity | | | |  |
| p value* | Tertile 3  72.02≤  N= 110 | Tertile 2  >50.84 <72.02  N= 110 | Tertile 1  ≤50.84  N= 110 | p value* | Tertile 3  73.15≤  N= 110 | Tertile 2  >51.52 <73.15  N= 110 | Tertile 1  ≤51.52  N= 110 | Variable |
| 0.252  0.376  0.197  <0.001  0.598  0.163  <0.001  0.480  0.917  0.19 | 111.59 (6.30)  257.93 (17.34)  290.91 (9.95)  90.32 (3.61)  35.67 (2.08)  17.83 (1.66)  496.42 (24.62)  12.84 (0.53)  88.16 (11.73)  162.94 (18.86) | 124.24 (4.19)  285.13 (11.46)  279.27 (6.64)  79.97 (2.41)  37.73 (1.39)  14.33 (1.11)  387.34 (16.43)  12.06 (0.35)  84.92 (7.82)  201.71 (12.63) | 123.15 (6.15)  298.03 (16.89)  260.90 (9.71)  64.93 (3.52)  36.36 (2.03)  12.52 (1.63)  299.10 (24.01)  12.12 (0.52)  89.48 (11.44)  219.62 (18.41) | 0.010  0.716  0.185  <0.001  0.638  0.798  <0.001  0.494  0.856  0.235 | 137.24 (6.39)  266.87 (17.80)  285.06 (10.19)  98.13 (3.60)  35.49 (2.13)  13.97 (1.71)  508.37 (25.10)  12.90 (0.54)  89.93 (12.01)  164.28 (19.30) | 118.88 (4.15)  282.9 (11.48)  283.85 (6.64)  75.98 (2.35)  37.62 (1.39)  15.37 (1.11)  382.02 (16.35)  12.35 (0.35)  83.97 (7.82)  201.60 (12.70) | 102.87 (6.24)  291.31 (17.37)  262.17 (9.95)  61.11 (3.52)  36.65 (2.08)  15.36 (1.68)  292.47 (24.51)  11.77 (0.53)  88.66 (11.72)  218.74 (18.92) | Grains (g)  Fruits (g)  Vegetables (g)  Meat and its products (g)  Beans (g)  Nuts and seeds (g)  Dairy products (g)  Fats (g)  Added sugar beverages(g)  Chocolate and snacks (g) |

SFA= saturated fatty acids, MUFA= monounsaturated fatty acids, and PUFA= polyunsaturated fatty acids. The p value is reported from covariance analysis, and the results are based on mean ± SD. All of the variables are adjusted for energy intake. *p value <0.05 shows a significant level of association

**Supplementary table 4.** Association between anthropometric indices and protein and fat quantity among primary school girls in Kerman

| Tertiles of macronutrient quantity (g) | | | | | | | | | |
| --- | --- | --- | --- | --- | --- | --- | --- | --- | --- |
| Tertiles of fat quantity | | | | Tertiles of protein quantity | | | | | |
| p value* | Tertile 3  72.02≤  N= 110 | Tertile 2  >50.84  <72.02  N= 110 | Tertile 1  ≤50.84  N= 110 | p value* | Tertile 3  73.15≤  N= 110 | Tertile 2  >51.52  <73.15  N= 110 | Tertile 1  ≤51.52  N= 110 | Variable | |
| <0.001  <0.001  0.276 | 23.46 (4.11)  23.09 (0.29)  22.50 (0.42) | 22.00 (3.42)  22.03 (0.28)  22.06 (0.28) | 20.46 (2.94)  20.79 (0.29)  21.35 (0.41) | <0.001  <0.001  0.634 | 23.48 (3.99)  23.07 (0.29)  22.32 (0.43) | 22.00 (3.55)  21.96 (0.28)  22.00 (0.28) | 20.44 (2.93)  20.88 (0.29)  21.60 (0.42) | Model 1^a^  Model 2^b^  Model 3^c^ | MUAC (cm) |
| <0.001  <0.001  0.102 | 0.67 (1.41)  0.62 (0.13)  0.31 (0.19) | 0.36 (1.33)  0.34 (0.12)  0.35 (0.12) | -0.48 (1.37)  -0.41 (0.13)  -0.12 (0.19) | <0.001  <0.001  0.485 | 0.71 (1.41)  0.65 (0.13)  0.31 (0.20) | 0.27 (1.36)  0.24 (0.13)  0.25 (0.13) | -0.42 (1.36)  -0.34 (0.13)  -0.01 (0.19) | Model 1^a^  Model 2^b^  Model 3^c^ | BAZ |
| 0.001  0.012  0.914 | 0.86 (1.08)  0.83 (0.11)  0.61 (0.17) | 0.64 (1.19)  0.61 (0.11)  0.62 (0.11) | 0.26 (1.32)  0.32 (0.11)  0.54 (0.16) | <0.001  0.001  0.068 | 0.806 (1.10)  0.77 (0.11)  0.57 (0.17) | 0.802 (1.20)  0.78 (0.11)  0.79 (0.11) | 0.16 (1.26)  0.21 (0.11)  0.40 (0.16) | Model 1^a^  Model 2^b^  Model 3^c^ | HAZ |
| 0.355  0.312  0.262 | 0.89 (1.33)  0.88 (0.98)  1.60 (1.49) | -0.96 (13.92)  -1.16 (0.91)  -1.16 (0.92) | -0.24 (1.21)  -0.05 (0.90)  -0.65 (1.31) | 0.357  0.364  0.288 | 0.90 (1.36)  0.88 (0.99)  1.72 (1.52) | -0.94 (13.92)  -1.03 (0.91)  -1.02 (0.91) | -0.26 (1.20)  -0.163 (0.89)  -0.87 (1.32) | Model 1^a^  Model 2^b^  Model 3^c^ | WAZ |

^a^Model 1 was resulted from one-way ANOVA, and the numbers are reported as mean ± SD. Model 1: crude.

^b^Model 2 was resulted from covariance analysis, and the numbers are reported as mean ± SE. p value <0.05 shows a significant level of association. Model 2: Adjusted for age, supplement use, parents' smoking, physical activity and socio-economic status.

^c^Model 3: Model 2+ energy intake
